# Hormonal Regulation of Urokinase‐ and Tissue‐Type Plasminogen Activator in Mouse Sertoli Cells

**DOI:** 10.1002/mrd.70012

**Published:** 2025-01-28

**Authors:** Sara Carosi, Federica Innocenti, Lucia Monaco, Gaia Laurenzi, Rossana Saracino, Rita Canipari, Elena Vicini

**Affiliations:** ^1^ Department of Anatomy, Histology, Forensic Medicine and Orthopedic, Section of Histology Sapienza University of Rome Rome Italy; ^2^ Department of Physiology and Pharmacology Sapienza University of Rome Rome Italy

**Keywords:** plasminogen activator, Sertoli cells, *uPA^−/−^* mice

## Abstract

A role for the plasminogen activator (PA) system has been postulated in mammalian gonads, considering the complex process of morphogenesis these organs undergo during their development. Our results show that mouse Sertoli cells under basal conditions produce both types of PA, tissue‐type PA (tPA) and urokinase‐type PA (uPA), and hormonal treatments increase the production of both enzymes. The increased enzyme secretion does not correlate with a parallel increase in their mRNAs. However, the proteolytic activity results from a balance between enzyme activity and inhibitors. Hormonal stimulation decreased the expression of the inhibitor PAI‐1, suggesting that the increase in proteolytic activity might depend on the decreased production of PAI‐1.

The expression of the two enzymes and their inhibitor depends on the seminiferous epithelium stage. We observed higher *uPA* mRNA levels at stages VII‐VIII and IX‐XII, *tPA* peaks at stages VII‐VIII, and *PAI‐1* mRNA levels decreased at stages VII‐VIII and IX‐XII.

The testes from mice lacking the *uPA* gene (*uPA* ^−/−^) presented statistically smaller sizes and weights. Histological analysis of *uPA* ^−/−^animals showed tubular morphology defects and atypical residual bodies (RB), suggesting a defect in Sertoli cell phagocytosis. Moreover, we show lower sperm concentration and motility in *uPA* ^−/−^ mice. These data suggested an effective deficiency of testicular development in the absence of uPA.

## 1 Introduction

Plasminogen activators (PAs) are serine proteases that cleave the proenzyme plasminogen present in plasma and extracellular fluids into the active protease plasmin.

In mammals, two forms of PA, urokinase‐type (uPA) and tissue type (tPA), have been characterized. The two PAs are encoded by different genes, but despite having distinct catalytic properties that may indicate distinct functions, they share the ability to cleave plasminogen to form the active protease plasmin (Dano et al. 1985). The presence of fibrin enhances the activity of tPA, whereas that of uPA is unaffected. This difference has been used to suggest that tPA physiological function is primarily fibrinolysis, whereas uPA may be involved in cell migration and tissue remodeling (Blasi 1993; Myohanen and Vaheri 2004). In addition, although tPA and uPA are secreted proteases, both can bind to the cell surface via specific cell surface receptors, thus protecting them from the inhibitory actions of the abundant plasma inhibitors (Dano et al. 1985; Danø et al. 2005). Moreover, two specific PA inhibitors have been identified: PA inhibitor‐1 (PAI‐1) and PA inhibitor‐2 (PAI‐2) (Andreasen et al. 1990).

A role for the PA system has been postulated in mammalian gonads, considering the complex process of morphogenesis these organs undergo during their development (Laurenzi et al. 2023; Le Magueresse‐Battistoni 2007). Tissue‐type PA, uPA, and PAI‐1 have been detected in the ovary. Their production by granulosa cells, as well as by other cell types present in the female gonads, has now been demonstrated in many mammals, although there are some differences in the type of PA produced (Canipari et al. 1987; Canipari and Strickland 1985; Epifano et al. 1994). Numerous hypotheses have been made about the difference of PA secreted by GCs of preovulatory follicles in the mouse, human, and rat ovary. The two enzymes could be interchangeable within the ovarian follicle of the three species, both being capable of producing plasmin; alternatively, there may be some required functions in the ovary of the different species for which tPA or uPA might be more suitable.

Sertoli cells and granulosa cells have a common embryonic origin, and both cell types secrete PAs under hormonal stimulation. Both enzymes have been detected in the rat testis, and are produced by Sertoli cells (Tolli et al. 1995) and myoid cells (Catizone et al. 2015). Under basal conditions, rat Sertoli cells secrete uPA, while in response to FSH stimulation, they increase tPA secretion and decrease uPA production (Tolli et al. 1995); PAI‐1 is secreted either by peritubular cells or by Sertoli cells (Le Magueresse‐Battistoni et al. 1998). Moreover, PAs are expressed in a stage‐dependent manner (Liu 2007; Vihko, Toppari, and Parvinen 1987). Therefore, it has been hypothesized that each of these molecules can be associated with different functions (Le Magueresse‐Battistoni 2007).

Starting from these observations and given the remarkable similarities between Sertoli cells and granulosa cells, this work aimed to study whether the differences in the production of PAs in the different species were also maintained in testes and their possible impact on testis functions.

This paper documents that mouse Sertoli cells produce both enzymes, with uPA highly regulated by cAMP. Moreover, the absence of uPA impairs spermatogenesis and testicular development.

## 2 Materials and Methods

### 2.1 Materials

Unless otherwise reported, chemicals were obtained from Sigma‐Aldrich.

### 2.2 Animal Models

C57BL/6 and *uPA*‐deficient (*uPA* ^−/−^) mice (Carmeliet et al. 1994) were housed under controlled temperature (25°C) and light conditions (12 h light/day) with access to food and water ad libitum. *uPA* ^−/−^ animals were backcrossed 10 times to mice of the C57BL/6 genetic background. All animal procedures were approved by the Local Ethics Committee for Animal Research (672/2018‐PR).

### 2.3 Genotyping

A little piece of mice tail was digested with 250 µL of NaOH (0.05 M) for 45 min at 95°C, afterwards was added 250 µL of TRIS‐HCl (0.5 M; pH 5.5) to stop the digestion process. Mice were genotyped by PCR using ReadyMIX Taq PCR Reaction Mix (Sigma‐Aldrich). The primers used are shown in Table 1.

**TABLE 1:** Sequence of oligonucleotides used as PCR primers.

*Gene*	Primers	Product length (bp)
*uPA*	Fw: 5′‐ATCGAAGGCCGCCCAACTCTGAGTGGGATTG‐3′ Rv: 5′‐TCCCAACAGCAGATCTCATGAATGACCC‐3′	509

Abbreviations: Fw, forward primer; Rv, reverse primer.

### 2.4 Sertoli Cell Preparation

Primary Sertoli cell cultures from 18 to 20‐day‐old C57BL/6 mice (Charles River, Como, Italy) were prepared as previously described (Tolli et al. 1995). Briefly, testes deprived of tunica albuginea, were subjected to sequential enzymatic digestions with trypsin (0.25%), collagenase (0.1%), and hyaluronidase (1 mg/mL) at 32°C for 30 min each. Between enzymatic treatment and washes, Sertoli cells were allowed to sediment by gravity. The final cell suspension was resuspended at a final concentration of 3 × 10^5^ viable cells/mL of serum‐free MEM supplemented with glutamine, nonessential amino acids, gentamicin, penicillin, and streptomycin, then cultured at 32°C in a 5% CO_2_ atmosphere. On the third day of culture, cells were treated with a hypotonic solution to remove contaminating germ cells (Galdieri et al. 1981). All treatments were started on the fourth day of culture and performed in medium supplemented with 0.1% BSA. At the end of incubation, culture fluids were collected and stored frozen until assayed for PA activity. The cells were washed extensively with fresh medium and further processed for RNA extraction.

### 2.5 Isolation of Seminiferous Tubules

Testes obtained from adult mice were deprived of tunica albuginea in a plate containing PBS. Under the stereomicroscope, with a magnification of 25–50×, the tubules were dissected from the interstitial tissue, and stages of the seminiferous epithelium were identified through the different patterns of light absorption by transillumination (Lamberti and Vicini 2014). Once the transillumination pattern of the different stages was identified, the tubules were cut into 2 mm segments and divided into three groups: stages II‐VI, VII‐VIII, and IX‐XII. The collected segments were lysed, and the RNA was extracted.

### 2.6 Rna Extraction, Reverse Transcription, and Real‐Time PCR

Total RNA was isolated using a silica gel‐based membrane spin column (RNeasy Kit, Qiagen S.p.A.) according to the manufacturer's instructions. Before use, ribonucleic acid integrity and purity were confirmed spectroscopically and by gel electrophoresis. Total RNA (2 μg) was reverse‐transcribed in a final volume of 20 μL using the M‐MLV Reverse Transcriptase kit (Invitrogen, Milan, Italy) according to the manufacturer's instructions. The presence of specific transcripts was evaluated by SYBR Green Real‐Time PCR on an Applied Biosystems 7500 Real‐Time PCR system as previously described (Innocenti et al. 2017). Each sample was normalized to its β‐actin content. Threshold cycle (Ct) values were used to calculate the fold changes in gene expression using the 2^−ΔΔCt^ method. The primers used are shown in Table 2.

**TABLE 2:** Sequence of oligonucleotides used as real‐time PCR primers.

*Gene*	Primers	Product length (bp)
*β‐Actin*	Fw: 5′‐TGACAGGATGCAGAAGGAGA‐3′ Rv: 5′‐GTACTTGCGCTCAGGAGGAG‐3′	82
*uPA*	Fw: 5′‐TAAAATGCTGTGTGCTGCGG‐3′ Rv: 5′‐CCGGGCTTGTTTTTCTCTGC‐3′	150
*tPA*	Fw: 5′‐TCGGGACACAGAAGAAACGG‐3′ Rv: 5′‐TTGTCTGCGTTGGCTCATCT‐3′	164
*PAI‐1*	Fw: 5′‐TCAGACAATGGAAGGGCAACA‐3′ Rv: 5′‐AGCTGCTCTTGGTCGGAAAG‐3′	169

Abbreviations: Fw, forward primer; Rv, reverse primer.

### 2.7 Assay for PA

Enzymatic activity of PA was assessed by the method of Shimada et al. (1981), as already described (Tolli et al. 1995). To characterize the type of PA present in the sample, the assay was performed in the presence or absence of 125 μM amiloride, a specific uPA inhibitor that does not affect tPA activity (Vassalli and Belin 1987). Values obtained in the presence of amiloride were considered dependent on tPA activity, while activity dependent on uPA was obtained by subtracting values due to tPA activity from total values. PA activity was expressed in terms of milli‐international units (mIU) with reference to a standard preparation of urokinase, and values were normalized to the value in milligrams of protein present in the culture dish. Protein concentrations were determined by the Bradford colorimetric assay (Bio‐Rad Laboratories) using BSA as standard.

### 2.8 Gel Electrophoresis and Zymography

For zymography of PA, culture media and cell lysates were separated by electrophoresis in 10% polyacrylamide slab gels in the presence of SDS (SDS‐PAGE) under nonreducing conditions (Laemmli 1970). PA was then visualized by placing the Triton X‐100‐washed gel on a casein‐agar‐plasminogen underlay as previously described (Catizone et al. 2012).

### 2.9 Cyclic AMP Assay

The amount of extracellular cAMP present in conditioned media was measured with a cAMP ELISA kit (Enzo Life Science, Euroclone S.p.A.) in accordance with manufacturer protocol. Values are normalized to milligrams of proteins present in the sample. Protein concentrations were determined by the Bradford colorimetric assay (Bio‐Rad Laboratories) using BSA as standard.

### 2.10 Morphological Studies

The testes from WT and *uPA* ^−/−^ animals of different ages (2.4, 4.5, and 6.5 months) were quickly removed, weighed, fixed in Bouin's liquid, embedded in paraffin, serially sectioned at 6 μm and stained with carmalum for 7 min at room temperature.

### 2.11 Sperm Preparation

Epididymis cauda were collected along with the vas deferens from adult WT and *uPA* ^−/−^ mice, transferred singularly into small Petri dishes, minced in M2 MEDIUM (Sigma‐Aldrich), and incubated for 15 min at 37°C (95% air and 5% CO_2_) to allow the release of sperm. Samples were diluted in a final volume of 1 mL, and sperm concentration was evaluated using a hemacytometer. In addition to the total number of spermatozoa, immobile spermatozoa were also counted to obtain a percentage of sperm motility.

### 2.12 Statistical Analysis

All experiments were repeated at least three times, and each experiment was performed at least in duplicate. Statistical analyses were performed using ANOVA followed by the Tukey–Kramer test for comparisons of multiple groups or a two‐tailed *t*‐test when comparing data derived from two groups. Values with *p* < 0.05 were considered statistically significant.

## 3 Results

### 3.1 Hormonal Regulation of PA Activity

We investigated the type of PA produced by mouse Sertoli cells in response to hormonal stimulation. Sertoli cells obtained from 18‐day‐old animals were treated for 18 h with 100 ng/mL FSH, alone (FSH) or together with 50–100 µM isobutyl methylxanthine (IBMX), a nonspecific phosphodiesterase inhibitor, and with increasing concentrations of dibutyryl‐cAMP (dbcAMP).

**FIGURE 1:**
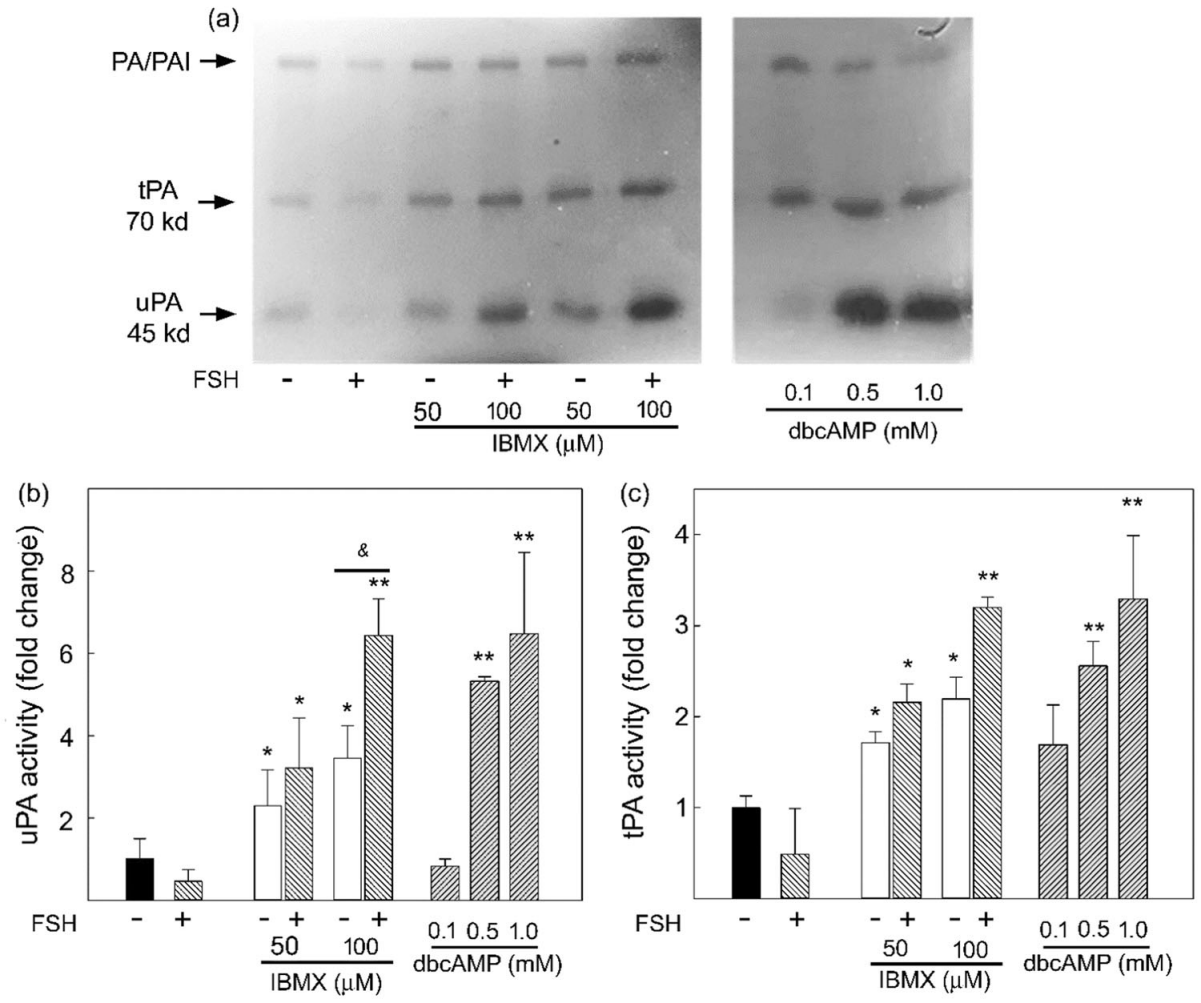
PA production by Sertoli cells in response to FSH and dbcAMP. Sertoli cell cultures from 18‐day‐old mice were prepared as described in Materials and Methods. On the fourth day of culture, cells were incubated for 18 h in MEM supplemented with 0.1% BSA with FSH (100 ng/mL) with or without 50 and 100 mM IBMX or increasing concentration of dbcAMP (0.1–1 mM). Medium was collected and assayed for PA activity at the end of the culture. Aliquots of conditioned media (20 mL) were analyzed by SDS‐PAGE followed by zymography or chromogenic substrate assay. (a) Representative zymography of five independent experiments. The photograph was taken after 18 h of incubation at 37°C. (b) uPA and (c) tPA activity analyzed by chromogenic substrate assay. Values represent mean ± SEM of five independent experiments and are expressed as fold change versus control (FSH‐), arbitrarily set at 1. Statistical analysis was performed using ANOVA followed by the Tukey–Kramer test. *p < 0.05 and **p < 0.01 versus control (FSH‐); & *p* < 0.05 versus respective IBMX alone.

Conditioned media were collected at the end of treatment, and PA activity was evidenced by zymography. As shown in Figure 1a, we observed the presence of the two types of PA. They have different molecular weights and can be distinguished by their different migration in the gel. The higher molecular weight band present in the gel represents the PA‐PAI inhibitor complex being partially activated under these experimental conditions. FSH alone does not induce Sertoli cell PA production. Instead, there is a significant stimulation of both enzyme production in response to dbcAMP, IBMX alone, and FSH in the presence of IBMX.

A chromogenic substrate assay was performed to quantify uPA and tPA in the Sertoli cell culture medium under the different culture conditions. PAs were expressed as fold change to the control arbitrarily set 1. As shown in Figure 1b,c, the uPA and tPA levels in the medium after the various treatments reflected those observed with zymography.

### 3.2 Hormonal Regulation of *tPA* and *uPA* mRNA Levels

To determine whether FSH and dbcAMP effects on PA activity were associated with changes in mRNA levels, total RNA was extracted from Sertoli cells cultured in the same conditions described above and analyzed by real‐time PCR.

**FIGURE 2:**
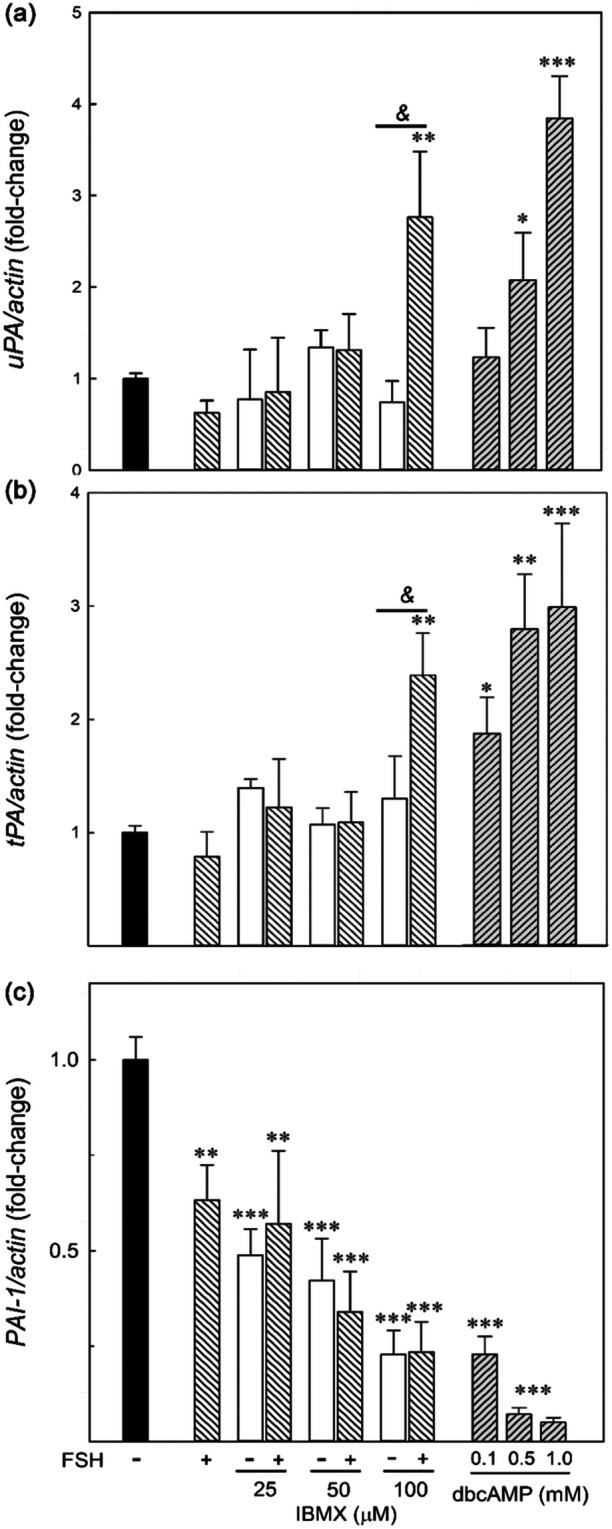
Total RNA, extracted from Sertoli cells obtained and cultured as described in Figure 1, was subject to real‐time PCR using primers specific for *uPA* (a), *tPA* (b), and *PAI*‐*1* (c). Each sample was normalized to its *b‐actin* content. Values represent mean ± SEM of five independent experiments and are expressed as fold change with respect to control (FSH‐), arbitrarily set at 1. Statistical analysis was performed using ANOVA followed by the Tukey–Kramer test. *p < 0.05, **p < 0.01, and ***p < 0.001 versus control (FSH‐); & *p* < 0.01 versus respective IBMX alone.

As shown in Figure 2a, FSH or IBMX alone did not stimulate either *uPA*‐ or *tPA*‐mRNA. Conversely, mRNA levels for both enzymes were significantly increased by stimulation with FSH and the highest dose of IBMX and dbcAMP at all concentrations (Figure 2a,b). To investigate PA regulation further, we evaluated the mRNA levels of *PAI‐1*, which is an important factor in regulating uPA and tPA enzymatic activity. The levels of *PAI‐1* mRNA significantly decreased with all treatments (Figure 2c).

### 3.3 Effect of FSH and IBMX on Sertoli Cell cAMP Production

Urokinase‐PA has been shown to be upregulated by cAMP in Sertoli cells (Bhattacharya et al. 2012) and Leydig cells (Demmouche and Tremblay 2022). Therefore, to evaluate whether the low response to FSH was due to a failure of cells to respond to FSH, we evaluated FSH stimulation's effect on the levels of cAMP in Sertoli cells.

Sertoli cells, obtained from 20‐day‐old mice, were cultured in the presence of 100 ng/mL FSH, 100 µM IBMX, and FSH + IBMX. At the end of the incubation, the cAMP levels present in the medium were evaluated by ELISA. As shown in Table 3, there was only a slight increase in cAMP production in response to IBMX alone. This production was significantly increased in the presence of FSH and further increased when, in addition to FSH, cells were stimulated with IBMX.

**TABLE 3:** Effect of FSH and IBMX on cAMP production by mouse Sertoli cells.

	cAMP (pmoles/mg proteins)	
	None	IBMX (100 µM)
Control	2.6 ± 0.73	6.9 ± 0.85*
FSH (100 ng/mL)	17.7 ± 0.64**	27.5 ± 0.574** ^,^ §

* *p* < 0.01

** *p* < 0.001 versus Control;

^§^ *p* < 0.001 versus FSH.

### 3.4 Stage‐Specific Expression of PA and PAI in Cultures Mouse Epithelium

To investigate the PA system's expression in the different stages of the seminiferous epithelium, we isolated stages I‐VI, VII‐VIII, and IX‐XI seminiferous tubule fragments by using transillumination microscopy. The mRNA levels of *uPA*, *tPA*, and *PAI*‐*1* were evaluated by Real‐time PCR.

**FIGURE 3:**
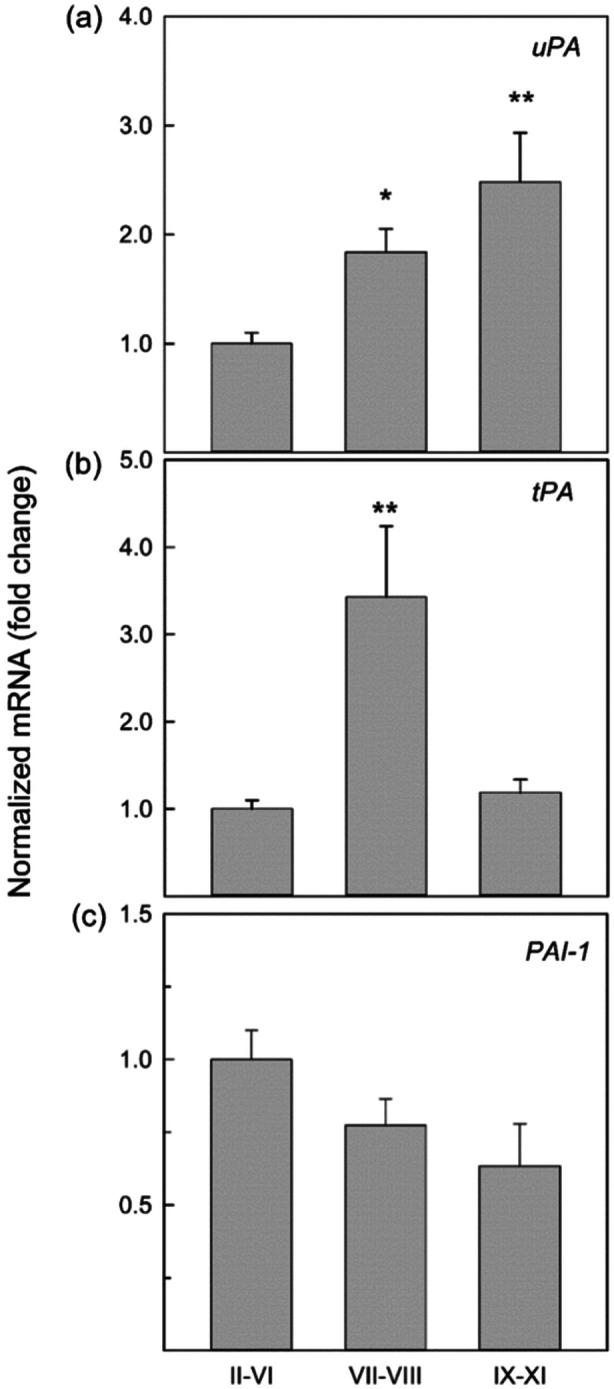
*uPA*, *tPA*, and *PAI*‐1 mRNA levels in staged seminiferous tubules isolated from adult mice. Total RNA, extracted from the different stages (II‐VI, VII‐VIII, IX‐XI), was subject to real‐time PCR using primers specific for *uPA* (a), *tPA* (b), and *PAI*‐*1* (c). Values represent mean ± SEM. of three independent experiments and are expressed as fold change normalized to stages II‐VI, arbitrarily set at 1 Statistical analysis was performed using ANOVA followed by the Tukey–Kramer test. *p < 0.05 and **p < 0.01 versus stages II‐VI.

As shown in Figure 3, the mRNA levels of *uPA* and *tPA* follow different trends. The *uPA* shows lower levels at stages II‐VI and a gradual increase in the transition from stages VII‐VIII to IX‐XI (Figure 3a), while *tPA* shows a peak in stages VII‐VIII (Figure 3b). *PAI*‐*1* mRNA levels are highest in stages II‐VI and gradually decrease at stages VII‐VIII and IX‐XI (Figure 3c).

### 3.5 Spermatogenesis in *uPA^−/−^* Mice

**FIGURE 4:**
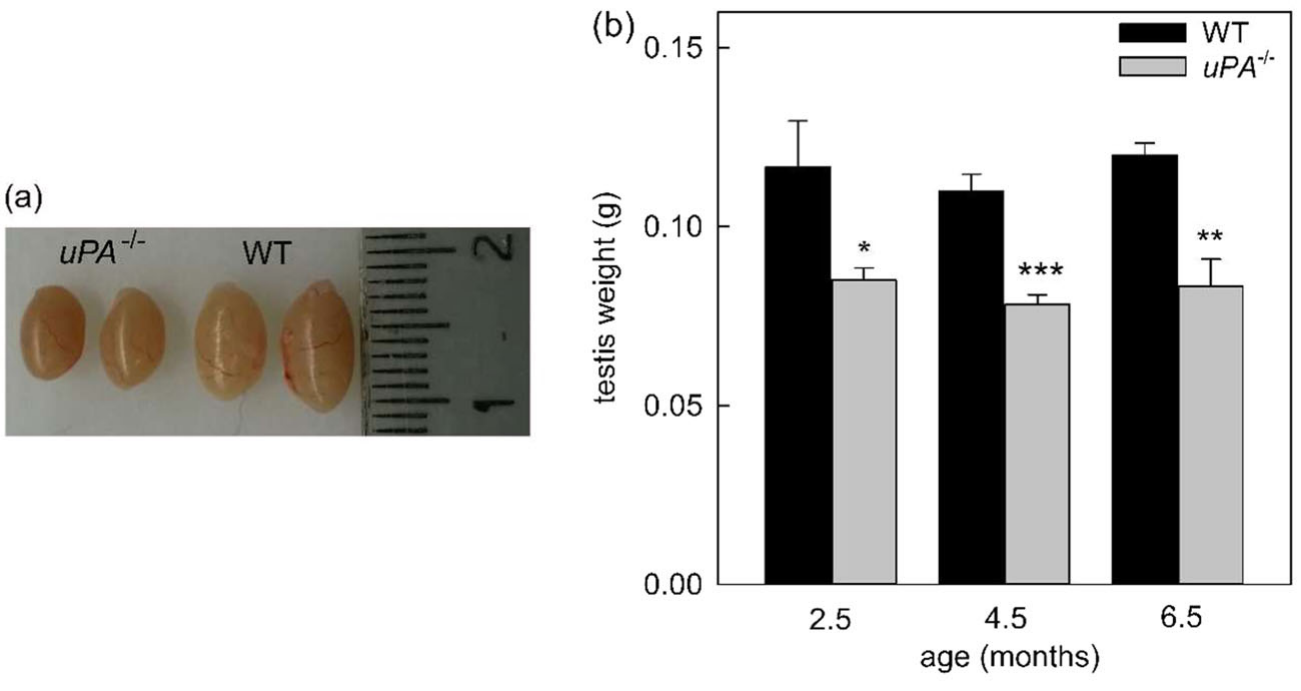
The absence of uPA impairs testis development. (a) Images of whole testes obtained from *uPA^−/−^* and WT 4.5‐month‐old mice (*n* = 6 each genotype). (b) Mean of the whole testis weight in WT and *uPA^−/−^* mice of different ages, with a total animal number of 3 age 2.5, 6 age 4.5, and 3 age 6.5 for each genotype. Statistical analysis was calculated by Student's *t*‐test. *p < 0.05, **p < 0.01, and ***p < 0.001 versus respective WT.

To have an initial assessment of the role of uPA in the process of spermatogenesis, we examined whether the deficiency of uPA in *uPA* knockout mice may alter testicular development and fertility. As shown in Figure 4, testes obtained from adult *uPA^−/−^* mice of different ages (2.5‐, 4.5‐, and 6.5‐month‐old) were smaller than the testes isolated from adult WT mice of the same age (Figure 4a,b).

**FIGURE 5:**
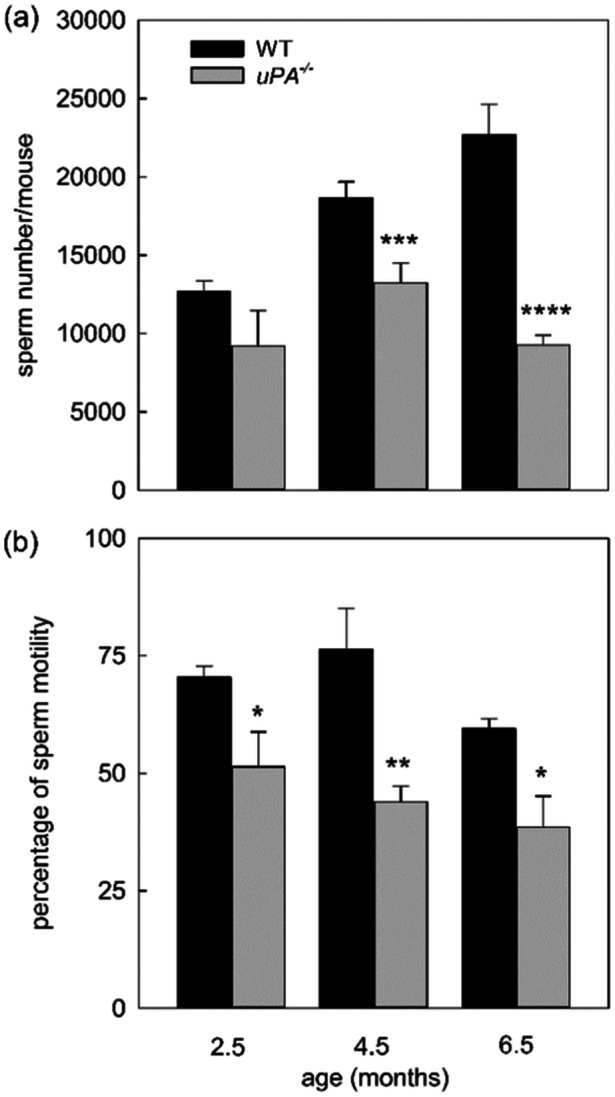
The absence of uPA impairs sperm number and motility. Mean of the number of spermatozoa (a) and mean of the percentage of sperm motility (b) in WT and *uPA^−/−^* mice of different ages. Results are expressed as number of spermatozoa and percentage of sperm motility per mouse with a total animal number of 3 age 2.5, 6 age 4.5, and 3 age 6.5 for each genotype. Statistical analysis was calculated by Student's *t*‐test. *p < 0.05, **p < 0.01, ***p < 0.005, and ****p < 0.001 versus respective WT.

We then examined spermatozoa recovered from the cauda epididymis of the same mice. We observed a reduction in both number and motility of spermatozoa in *uPA^−/−^* animals compared to WT. The decrease in the sperm number with respect to WT animals, was more evident in older animals (Figure 5a,b).

### 3.6 Morphological Evaluation of Seminiferous Tubules

**FIGURE 6:**
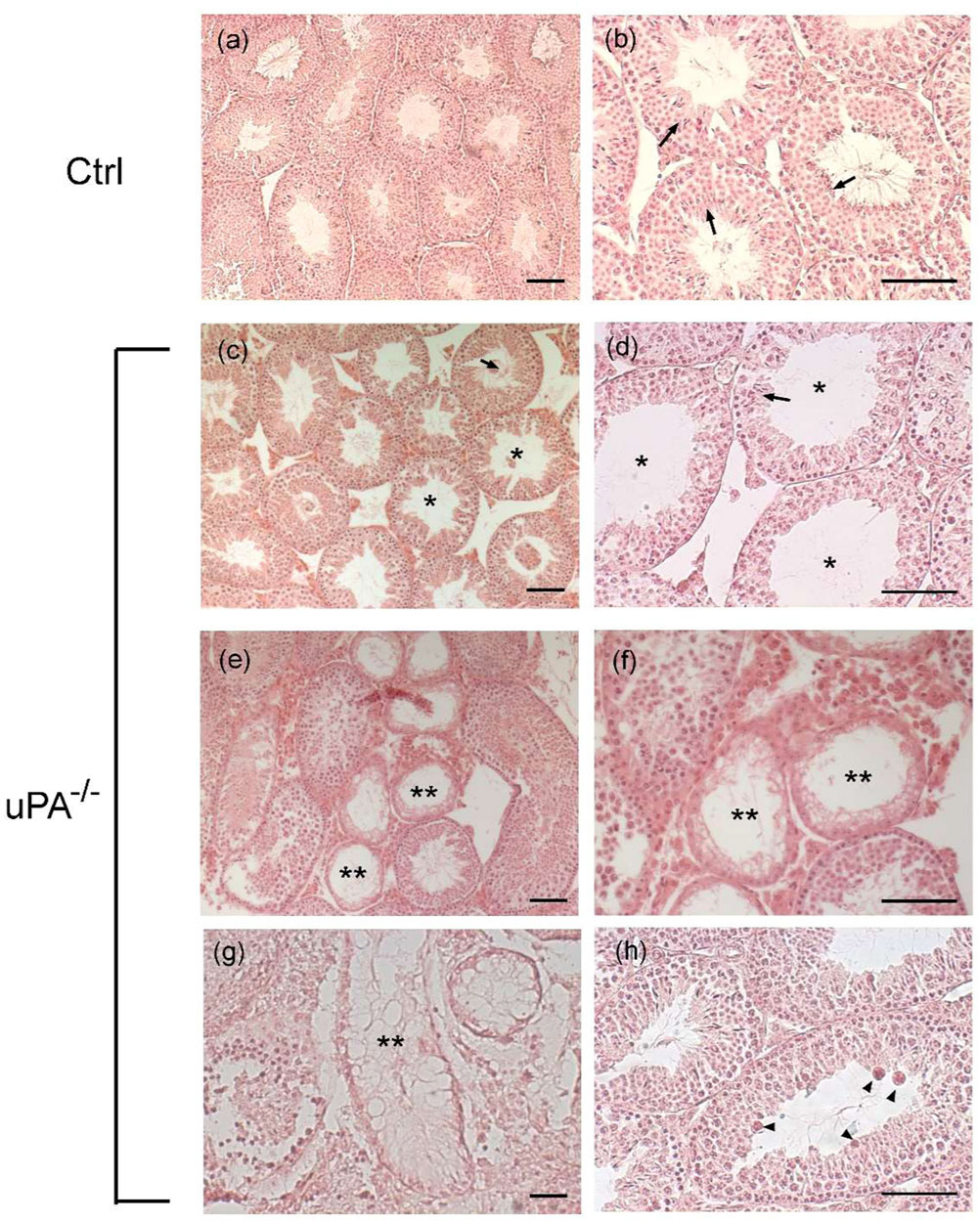
Photomicrographs of sections of testes from WT (a, b), *uPA^−/−^* (c–h) mice. Arrows: spermatozoa; *: tubules with enlarged lumen; **: Sertoli cell‐only tubules; arrowheads: residual bodies. Scale bar = 100 μm.

We then examined how the absence of uPA affects testis morphology in 4.5‐month‐old animals. The histological observations on testis of WT animals showed seminiferous tubules characterized by a correct structure with a well‐defined lumen and complete spermatogenesis (Figure 6). The testis of the *uPA^−/−^* animals showed several degrees of damage. Increased luminal diameter of seminiferous tubules, atypical residual bodies, thinner epithelium with less germ cells, Sertoli cell‐only tubules (Figure 6). However, in the same animal group, some seminiferous tubules were characterized by seemingly normal morphology.

## 4 Discussion

Previous studies on the PA produced by granulosa cells obtained from mouse, human, and rat ovaries have shown that in the ovaries of these species, the type of PA produced by granulosa cells is species‐specific (Canipari et al. 1987; Epifano et al. 1994). In response to gonadotropins, rat granulosa cells secrete tPA and decrease the production of uPA (Canipari and Strickland 1986). Identical results are obtained with rat Sertoli cells where FSH induces tPA production while inhibiting uPA (Tolli et al. 1995). Conversely, in mouse granulosa cells, gonadotropin stimulation induces uPA secretion (Canipari et al. 1987). Here, we show that, in basal conditions, mouse Sertoli cells produce low levels of both types of PAs and that FSH alone did not affect uPA and tPA production. However, the co‐treatment with FSH and IBMX or dibutyl cAMP stimulated the production of both PAs. These data suggested the involvement of the cAMP pathway in the *uPA* and *tPA* gene modulation, as already shown in the rat (Macchione et al. 2000) and mouse granulosa cells (Canipari et al. 1987) and in rat Sertoli cells (Tolli et al. 1995). Our data indicate that, like in the ovary, hormonal stimulation in the testis activates different types of PAs across various species. However, although these PAs have distinct catalytic properties, they ultimately catalyze the cleavage of the same bond in plasminogen with plasmin formation, allowing their functions to be interchangeable (Danø et al. 2005).

To gain more insight into PA production regulation, we evaluated if the control was at the mRNA level. Our analysis of *tPA*‐ and *uPA*‐mRNA expression revealed that the levels of mRNAs did not mirror those of enzymatic activity. In fact, despite the increased PA‐activity observed in the medium in response to FSH and IBMX stimulation, FSH alone or in the presence of IBMX, as well as IBMX alone, had no effect on *uPA*‐ or *tPA*‐mRNA, apart from FSH plus 100 μM IBMX. This lack of effect of FSH on PA expression could be due to low levels of cAMP obtained by stimulating with FSH alone insufficient to stimulate PAs (Table 3).

The enzymatic activity in the medium is a balance of PA activity and PAI inhibitory action (Le Magueresse‐Battistoni 2007). The high molecular weight lytic band in the zymography evidenced the presence of a PA inhibitor in Sertoli cell‐conditioned media. From the analysis at the mRNA levels, we observed that the stimulation with FSH alone caused a significant decrease in *PAI‐1*‐mRNA levels. The same decrease of *PAI‐1* in response to FSH has also been observed in the rat testis (Le Magueresse‐Battistoni et al. 1998). The addition of IBMX to FSH further decreased *PAI‐1* expression. These results suggested that the control of total PA enzymatic activity in response to IBMX or FSH plus IBMX was not dependent on an increase in the levels of their mRNA but through a decreased inhibition by PAI‐1 present in the medium. Therefore, after hormonal stimulation, a decrease in the *PAI‐1*‐mRNA corresponds to an increase in the PA enzymatic activity present in the medium. Moreover, the data obtained treating Sertoli cells with dbcAMP suggested that FSH stimulates *uPA* and *tPA* expression and inhibits *PAI‐1* expression through the cAMP pathway.

A role for these proteases has been suggested in the continuous remodeling of the rat seminiferous epithelium taking place during the release of the preleptotene spermatocytes from the basement membrane (Russell 1977), spermiation (Leblond and Clermont 1952), detachment of residual bodies from the mature spermatids (Morales, Clermont, and Nadler 1986), and their phagocytosis by Sertoli cells (Sigillo et al. 1998). The secretion of PAs by rat and mouse seminiferous epithelial cells is stage‐specific. It is preferentially observed at stages VII and VIII and IX‐XII of the cycle, suggesting that PAs may be involved during critical stages of spermatogenesis (Fritz, Tung, and Ailenberg 1993; Lacroix, Parvinen, and Fritz 1981; Liu 2007). Ailenberg and Fritz (1988) suggested that PAs play a role in breaking the tight junctions between adjacent rat Sertoli cells that can be observed at stages VII and VIII of the cycle. This mechanism would allow the migration of spermatocytes toward the adluminal compartment (Ailenberg and Fritz 1988). Moreover, in a two‐chamber model system, following stimulation by FSH or dbcAMP, lower integrity was observed in the barrier formed by the Sertoli cells (Ailenberg and Fritz 1989; Catizone, Ricci, and Galdieri 2008). Conversely, the production of protease inhibitors by Sertoli cells in the adluminal compartment can modulate the net protease activity within the seminiferous tubule at defined stages, such as during the process of translocation and spermiation, therefore maintaining the integrity of the Sertoli cell barrier (Le Magueresse‐Battistoni 2007; Le Magueresse‐Battistoni et al. 1998).

We further investigated this aspect and observed higher *uPA*‐mRNA levels at stages VII‐VIII and IX‐XI, while *tPA*‐mRNA peaks at stages VII‐VIII. Conversely, *PAI‐1*‐mRNA levels showed a decreasing trend at stages VII‐VIII and IX‐XI. The decline in *PAI‐1* expression paralleled by an increase in PA expression at later stages may result in an increase in PA's activity in the second part of the cycle. Interestingly, immunostaining for a2‐macroglobulin, a nonspecific protease inhibitor, has been found at stages I‐VI (Wong and Cheng 2005), where we found higher levels of *PAI‐1* expression.

Here, we present evidence that in immature Sertoli cells, the expression levels of PAs are under the control of the cAMP pathway. However, the analysis of PA expression levels during the seminiferous epithelium cycle shows that *uPA* and *tPA* expression at stages II‐VI is lower compared to subsequent stages. It is important to emphasize that even though the highest response in terms of cAMP levels in Sertoli cells occurs predominately in stages I‐V of the cell cycle (Kangasniemi et al. 1990; Parvinen 1982), PAs are also influenced by several other factors locally produced in the testis (Le Magueresse‐Battistoni 2007). Among these are retinoic acid (RA), Hepatocyte Growth Factor (HGF), and tumor necrosis factor‐alfa (TNF‐α). RA levels are highest at stage VIII‐XI of the seminiferous epithelial cycle (Hogarth et al. 2015). In rat Sertoli cells, RA has been shown to inhibit transcription of the *uPA* (Canipari and Galdieri 2000). Moreover, RA has been shown to stimulate *PA* expression in the rat testis in a stage‐dependent manner (Vihko, Toppari, and Parvinen 1987). HGF in adult rats is maximally expressed at stages VI‐VII of the cycle, when germ cells traverse the blood‐testis‐barrier, and when myoid cell contraction occurs, and declines in the subsequent stages IX‐VI (Catizone et al. 2012). HGF has been shown to stimulate the expression of *uPA* in rat myoid cells, and myoid cell contraction is abrogated by uPA inhibitors (Catizone et al. 2015). TNF‐α is maximally expressed at stages X and XI of the cycle by pachytene spermatocytes and round spermatids (De et al. 1993), and it has been demonstrated to induce the expression of PAI‐1 in peritubular cells (Le Magueresse‐Battistoni et al. 1997). These findings suggest that several factors contribute to the regulation of the expression of *PAs* and *PAI* at the different stages of the seminiferous epithelium cycle.

In the literature, there is some evidence that links uPA with spermatogenesis. The urokinase receptor was identified at the point of contact between the Sertoli and germ cells, indicating that the proteolysis involving plasminogen could occur near germ cells (Le Magueresse‐Battistoni 2007). In postnatal rat testis, the expression of *uPA* and *uPAR* increases with the proceedings of the first wave of sperm maturation, and the highest levels were observed at postnatal Day 35, when round spermatids are transformed to elongate spermatids and began spermiation (Huang et al. 2007). Urokinase‐PA is involved in the modulation of occludin levels in endothelial cells (Behzadian et al. 2003), and in the testis, it is closely related to the tight junctions of the blood‐testis barrier (Tian et al. 2007; Wong et al. 2000). Moreover, it has been demonstrated that HGF strongly increases the amount of uPA secreted by the tubules (Catizone et al. 2012). This secreted uPA, in turn, promotes the activation of inactive TGF‐β (Catizone, Ricci, and Galdieri 2008; Nunes, Shapiro, and Rifkin 1995; Odekon, Blasi, and Rifkin 1994), and the active TGF‐β can perturb Sertoli cell tight junctions (Lui, Lee, and Cheng 2003). Finally, it has been observed reduced fertility in mice after subcutaneous injection of anti‐human uPA antibodies (Ding et al. 2018; Qin et al. 2015) and after uPA‐inhibition by lentiviral RNA interference (Zhao et al. 2017).

The present study shows that *uPA^−/−^* mice display smaller testis with altered seminiferous tubular morphology and lower sperm concentration and motility. Moreover, the presence of atypical residual bodies (RB) in *uPA^−/−^* animals suggested a defect in RB phagocytosis by Sertoli cells. A correlation has been shown between RB phagocytosis, uPA secretion by Sertoli cells, and the initiation of a new wave of spermatogenesis (Sigillo et al. 1998). Therefore, impaired phagocytosis and uPA production may lead to defects in germ cell production. Our data are in line with the data showing reduced sperm concentration, sperm viability, and sperm motility when uPA is experimentally inhibited in the mouse testis (Qin et al. 2015; Zhao et al. 2017). Altogether, these data suggested that uPA plays a role in the production and correct maturation of spermatozoa.

In conclusion, similar to what is observed in the ovary, Sertoli cells in the testes from various species perform similar functions by expressing functionally related but genetically distinct enzymes. Although hormone regulation and production vary across species, suggesting that each enzyme may have unique functions, it is important to note that despite differences in their catalytic properties, these enzymes catalyze the same bond cleavage in plasminogen. Studies in gene‐deficient mice further indicate that PAs can substitute for one another (Danø et al. 2005). These findings highlight the intricate regulatory mechanisms underlying PA production and emphasize the need for further research in this area. Understanding the expression patterns of *tPA* and *uPA* in Sertoli cells across species may help determine the function of these enzymes in testicular physiology.

## Author Contributions

**Sara Carosi:** methodology, investigation. **Federica Innocenti:** methodology, investigation. **Lucia Monaco:** conceptualization, investigation, validation, formal analysis, methodology. **Gaia Laurenzi:** Investigation. **Rossana Saracino:** methodology, investigation. **Rita Canipari:** supervision, data curation, writing–review and editing, writing–original draft, funding acquisition, project administration, validation, conceptualization, resources, formal analysis. **Elena Vicini:** conceptualization, writing–review and editing, resources, validation, data curation.

## Conflicts of Interest

The authors declare no conflicts of interest.

## Data Availability

The data that support the findings of this study are available from the corresponding author upon reasonable request.

## References

[mrd70012-bib-0001] Ailenberg, M. , and I. B. Fritz . 1988. “Control of Levels of Plasminogen Activator Activity Secreted by Sertoli Cells Maintained in a Two‐Chamber Assembly.” Endocrinology 122: 2613–2618. 10.1210/endo-122-6-2613. 2453345

[mrd70012-bib-0002] Ailenberg, M. , and I. B. Fritz . 1989. “Influences of Follicle‐Stimulating Hormone, Proteases, and Antiproteases on Permeability of the Barrier Generated by Sertoli Cells in a Two‐Chambered Assembly.” Endocrinology 124: 1399–1407. 10.1210/endo-124-3-1399. 2465139

[mrd70012-bib-0003] Andreasen, P. A. , B. Georg , L. R. Lund , A. Riccio , and S. N. Stacey . 1990. “Plasminogen Activator Inhibitors: Hormonally Regulated Serpins.” Molecular and Cellular Endocrinology 68: 1–19. 10.1016/0303-7207(90)90164-4. 2105900

[mrd70012-bib-0004] Behzadian, M. A. , L. J. Windsor , N. Ghaly , G. Liou , N. T. Tsai , and R. B. Caldwell . 2003. “Vegf‐Induced Paracellular Permeability in Cultured Endothelial Cells Involves Urokinase and Its Receptor.” The FASEB Journal 17: 752–754. 10.1096/fj.02-0484fje. 12594181

[mrd70012-bib-0005] Bhattacharya, I. , B. S. Pradhan , K. Sarda , M. Gautam , S. Basu , and S. S. Majumdar . 2012. “A Switch in Sertoli Cell Responsiveness to Fsh May Be Responsible for Robust Onset of Germ Cell Differentiation During Prepubartal Testicular Maturation in Rats.” American Journal of Physiology‐Endocrinology and Metabolism 303: E886–E898. 10.1152/ajpendo.00293.2012. 22850685

[mrd70012-bib-0006] Blasi, F. 1993. “Urokinase and Urokinase Receptor: A Paracrine/Autocrine System Regulating Cell Migration and Invasiveness.” BioEssays 15: 105–111. 10.1002/bies.950150206. 8385942

[mrd70012-bib-0007] Canipari, R. , and M. Galdieri . 2000. “Retinoid Modulation of Plasminogen Activator Production in Rat Sertoli Cells.” Biology of Reproduction 63: 544–550. 10.1095/biolreprod63.2.544. 10906063

[mrd70012-bib-0008] Canipari, R. , M. L. O'Connell , G. Meyer , and S. Strickland . 1987. “Mouse Ovarian Granulosa Cells Produce Urokinase‐Type Plasminogen Activator, Whereas the Corresponding Rat Cells Produce Tissue‐Type Plasminogen Activator.” Journal of Cell Biology 105: 977–981. 10.1083/jcb.105.2.977. 3040774 PMC2114761

[mrd70012-bib-0009] Canipari, R. , and S. Strickland . 1985. “Plasminogen Activator in the Rat Ovary. Production and Gonadotropin Regulation of the Enzyme in Granulosa and Thecal Cells.” Journal of Biological Chemistry 260: 5121–5125. 10.1016/S0021-9258(18)89187-6. 3921542

[mrd70012-bib-0010] Canipari, R. , and S. Strickland . 1986. “Studies on the Hormonal Regulation of Plasminogen Activator Production in the Rat Ovary.” Endocrinology 118: 1652–1659. 10.1210/endo-118-4-1652. 3081331

[mrd70012-bib-0011] Carmeliet, P. , L. Schoonjans , L. Kieckens , et al. 1994. “Physiological Consequences of Loss of Plasminogen Activator Gene Function in Mice.” Nature 368: 419–424. 10.1038/368419a0. 8133887

[mrd70012-bib-0013] Catizone, A. , G. Ricci , M. Caruso , F. Ferranti , R. Canipari , and M. Galdieri . 2012. “Hepatocyte Growth Factor (HGF) Regulates Blood‐Testis Barrier (BTB) in Adult Rats.” Molecular and Cellular Endocrinology 348: 135–146. 10.1016/j.mce.2011.07.050. 21843593

[mrd70012-bib-0012] Catizone, A. , G. Ricci , M. Caruso , et al. 2015. “HGF Modulates Actin Cytoskeleton Remodeling and Contraction in Testicular Myoid Cells.” Biomedicines 3: 89–109. 10.3390/biomedicines3010089. 28536401 PMC5344232

[mrd70012-bib-0014] Catizone, A. , G. Ricci , and M. Galdieri . 2008. “Hepatocyte Growth Factor Modulates Sertoli‐Sertoli Tight Junction Dynamics.” Journal of Cellular Physiology 216: 253–260. 10.1002/jcp.21400. 18265003

[mrd70012-bib-0015] Dano, K. , A. Andreasen , J. Grondahl‐Hansen , P. Kristensen , L. S. Nielsen , and L. Skriver . 1985. “Plasminogen Activators, Tissue Degradation and Cancer.” Advances in Cancer Research 44: 139–266. 10.1016/S0065-230X(08)60028-7. 2930999

[mrd70012-bib-0016] Danø, K. , N. Behrendt , G. Høyer‐Hansen , et al. 2005. “Plasminogen Activation and Cancer.” Thrombosis and Haemostasis 93: 676–681. 10.1160/th05-01-0054. 15841311

[mrd70012-bib-0017] De, S. K. , H. L. Chen , J. L. Pace , J. S. Hunt , P. F. Terranova , and G. C. Enders . 1993. “Expression of Tumor Necrosis Factor‐Alpha in Mouse Spermatogenic Cells.” Endocrinology 133: 389–396. 10.1210/endo.133.1.8319585. 8319585

[mrd70012-bib-0018] Demmouche, Z. B. , and J. J. Tremblay . 2022. “The Urokinase‐Type Plasminogen Activator Contributes to cAMP‐Induced Steroidogenesis in MA‐10 Leydig Cells.” Endocrines 3: 460–475. 10.3390/endocrines3030037.

[mrd70012-bib-0019] Ding, X. , H. Li , Y. Li , D. Huang , and C. Xiong . 2018. “Two B‐Cell Epitope Vaccines Based on uPA Effectively Inhibit Fertility in Male Mice.” Vaccine 36: 2612–2618. 10.1016/j.vaccine.2018.03.071. 29631885

[mrd70012-bib-0020] Epifano, O. , M. Riminucci , C. Manna , et al. 1994. “In Vitro Production of Plasminogen Activator by Human Granulosa Cells.” Journal of Clinical Endocrinology and Metabolism 78: 174–179. 10.1210/jcem.78.1.8288701. 8288701

[mrd70012-bib-0021] Fritz, I. B. , P. S. Tung , and M. Ailenberg . 1993. “Proteases and Antiproteases in the Seminiferous Tubules.” In The Sertoli Cell, edited by L. D. Russell and M. D. Griswold , 217–235. Clearwater, Florida: Cache River Press.

[mrd70012-bib-0022] Galdieri, M. , E. Ziparo , F. Palombi , M. A. Russo , and M. Stefanini . 1981. “Pure Sertoli Cell Cultures: A New Model for the Study of Somatic‐Germ Cell Interactions.” Journal of Andrology 2: 249–254. 10.1002/J.1939-4640.1981.TB00625.X.

[mrd70012-bib-0023] Hogarth, C. A. , S. Arnold , T. Kent , D. Mitchell , N. Isoherranen , and M. D. Griswold . 2015. “Processive Pulses of Retinoic Acid Propel Asynchronous and Continuous Murine Sperm Production.” Biology of Reproduction 92: 37. 10.1095/biolreprod.114.126326. 25519186 PMC4326729

[mrd70012-bib-0024] Huang, D. H. , H. Zhao , Y. H. Tian , H. G. Li , X. F. Ding , and C. L. Xiong . 2007. “Gene Expression Changes of Urokinase Plasminogen Activator and Urokinase Receptor in Rat Testes at Postnatal Stages.” Asian Journal of Andrology 9: 679–683. 10.1111/j.1745-7262.2007.00272.x. 17712486

[mrd70012-bib-0025] Innocenti, F. , L. Cerquetti , S. Pezzilli , et al. 2017. “Effect of Mitotane on Mouse Ovarian Follicle Development and Fertility.” Journal of Endocrinology 234: 29–39. 10.1530/JOE-17-0203. 28450646

[mrd70012-bib-0026] Kangasniemi, M. , A. Kaipia , J. Toppari , A. Perheentupa , I. Huhtaniemi , and M. Parvinen . 1990. “Cellular Regulation of Follicle‐Stimulating Hormone (FSH) Binding in Rat Seminiferous Tubules.” Journal of Andrology 11: 336–343. 2120166

[mrd70012-bib-0027] Lacroix, M. , M. Parvinen , and I. B. Fritz . 1981. “Localization of Testicular Plasminogen Activator in Discrete Portions (Stages VII and VIII) of the Seminiferous Tubule.” Biology of Reproduction 25: 143–146. 10.1095/biolreprod25.1.143. 7197172

[mrd70012-bib-0028] Laemmli, U. K. 1970. “Cleavage of Structural Proteins During the Assembly of the Head of Bacteriophage T4.” Nature 227: 680–685. 5432063 10.1038/227680a0

[mrd70012-bib-0029] Lamberti, D. , and E. Vicini . 2014. “Promoter Analysis of the Gene Encoding GDNF in Murine Sertoli Cells.” Molecular and Cellular Endocrinology 394: 105–114. 10.1016/j.mce.2014.07.006. 25025809

[mrd70012-bib-0030] Laurenzi, G. , V. Fedeli , and R. Canipari . 2024. “Decreased Fertility in Female Mice Lacking Urokinase Plasminogen Activator.” Reproductive Biology 24: 100840. 10.1016/j.repbio.2023.100840. 38113659

[mrd70012-bib-0031] Leblond, C. P. , and Y. Clermont . 1952. “Definition of the Stages of the Cycle of the Seminiferous Epithelium in the Rat.” Annals of the New York Academy of Sciences 55: 548–573. 10.1111/j.1749-6632.1952.tb26576.x. 13139144

[mrd70012-bib-0032] Liu, Y. X. 2007. “Involvement of Plasminogen Activator and Plasminogen Activator Inhibitor Type 1 in Spermatogenesis, Sperm Capacitation, and Fertilization.” Seminars in Thrombosis and Hemostasis 33: 029–040. 10.1055/s-2006-958459. 17253187

[mrd70012-bib-0033] Lui, W. Y. , W. M. Lee , and C. Y. Cheng . 2003. “TGF‐βs: Their Role in Testicular Function and Sertoli Cell Tight Junction Dynamics.” International Journal of Andrology 26: 147–160. 10.1046/j.1365-2605.2003.00410.x. 12755993

[mrd70012-bib-0034] Macchione, E. , O. Epifano , M. Stefanini , D. Belin , and R. Canipari . 2000. “Urokinase Redistribution From the Secreted to the Cell‐Bound Fraction in Granulosa Cells of Rat Preovulatory Follicles.” Biology of Reproduction 62: 895–903. 10.1095/biolreprod62.4.895. 10727258

[mrd70012-bib-0035] Le Magueresse‐Battistoni, B. 2007. “Serine Proteases and Serine Protease Inhibitors in Testicular Physiology: The Plasminogen Activation System.” Reproduction 134: 721–729. 10.1530/rep-07-0114. 18042629

[mrd70012-bib-0036] Le Magueresse‐Battistoni, B. , G. Pernod , L. Kolodié , A. M. Morera , and M. Benahmed . 1997. “Tumor Necrosis Factor‐α Regulates Plasminogen Activator Inhibitor‐1 in Rat Testicular Peritubular Cells*.” Endocrinology 138: 1097–1105. 10.1210/endo.138.3.4963. 9048615

[mrd70012-bib-0037] Le Magueresse‐Battistoni, B. , G. Pernod , F. Sigillo , L. Kolodié , and M. Benahmed . 1998. “Plasminogen Activator inhibitor‐1 Is Expressed in Cultured Rat Sertoli Cells.” Biology of Reproduction 59: 591–598. 10.1095/biolreprod59.3.591. 9716558

[mrd70012-bib-0038] Morales, C. , Y. Clermont , and N. J. Nadler . 1986. “Cyclic Endocytic Activity and Kinetics of Lysosomes in Sertoli Cells of the Rat.” Biology of Reproduction 34: 207–218. 10.1095/biolreprod34.1.207. 3955137

[mrd70012-bib-0039] Myohanen, H. , and A. Vaheri . 2004. “Regulation and Interactions in the Activation of Cell‐Associated Plasminogen.” Cellular and Molecular Life Sciences 61: 2840–2858. 10.1007/s00018-004-4230-9. 15558213 PMC11924493

[mrd70012-bib-0040] Nunes, I. , R. L. Shapiro , and D. B. Rifkin . 1995. “Characterization of Latent TGF‐Beta Activation by Murine Peritoneal Macrophages.” The Journal of Immunology 155: 1450–1459. 7636210

[mrd70012-bib-0041] Odekon, L. E. , F. Blasi , and D. B. Rifkin . 1994. “Requirement for Receptor‐Bound Urokinase in Plasmin‐Dependent Cellular Conversion of Latent TGF‐β to TGF‐Β.” Journal of Cellular Physiology 158: 398–407. 10.1002/jcp.1041580303. 8126064

[mrd70012-bib-0042] Parvinen, M. 1982. “Regulation of the Seminiferous Epithelium.” Endocrine Reviews 3: 404–417. 6295753 10.1210/edrv-3-4-404

[mrd70012-bib-0043] Qin, Y. , Y. Han , C. L. Xiong , H. G. Li , L. Hu , and L. Zhang . 2015. “Urokinase‐Type Plasminogen Activator: A New Target for Male Contraception?” Asian Journal of Andrology 17: 269–273. 10.4103/1008-682x.143316. 25578931 PMC4650482

[mrd70012-bib-0044] Russell, L. 1977. “Movement of Spermatocytes From the Basal to the Adluminal Compartment of the Rat Testis.” American Journal of Anatomy 148: 313–328. 10.1002/aja.1001480303. 857632

[mrd70012-bib-0045] Shimada, H. , T. Mori , A. Takada , et al. 1981. “Use of Chromogenic Substrate S‐2251 for Determination of Plasminogen Activator in Rat Ovaries.” Thrombosis and Haemostasis 46: 507–510. 7197812

[mrd70012-bib-0046] Sigillo, F. , G. Pernod , L. Kolodie , M. Benahmed , and B. Le Magueresse‐Battistoni . 1998. “Residual Bodies Stimulate Rat Sertoli Cell Plasminogen Activator Activity.” Biochemical and Biophysical Research Communications 250: 59–62. 10.1006/bbrc.1998.9264. 9735331

[mrd70012-bib-0047] Tian, Y. H. , C. L. Xiong , H. Wan , et al. 2007. “Inhibition of the Urokinase‐Type Plasminogen Activator by Triplex‐Forming Oligonucleotides in Rat Sertoli Cells: A New Contraceptive Alternative?” Oligonucleotides 17: 174–188. 10.1089/oli.2006.0068. 17638522

[mrd70012-bib-0048] Tolli, R. , L. Monaco , P. DiBonito , and R. Canipari . 1995. “Hormonal Regulation of Urokinase‐ and Tissue‐Type Plasminogen Activator in Rat Sertoli Cells.” Biology of Reproduction 53: 193–200. 10.1095/biolreprod53.1.193. 7545441

[mrd70012-bib-0049] Vassalli, J. D. , and D. Belin . 1987. “Amiloride Selectively Inhibits the Urokinase‐Type Plasminogen Activator.” FEBS Letters 214: 187–191. 10.1016/0014-5793(87)80039-x. 3106085

[mrd70012-bib-0050] Vihko, K. K. , J. Toppari , and M. Parvinen . 1987. “Stage‐Specific Regulation of Plasminogen Activator Secretion in the Rat Seminiferous Epithelium.” Endocrinology 120: 142–145. 10.1210/endo-120-1-142. 3023024

[mrd70012-bib-0051] Wong, C. C. S. , S. S. W. Chung , J. Grima , et al. 2000. “Changes in the Expression of Junctional and Nonjunctional Complex Component Genes When Inter‐Sertoli Tight Junctions Are Formed In Vitro.” Journal of Andrology 21: 227–237. 10714817

[mrd70012-bib-0052] Wong, C. H. , and C. Y. Cheng . 2005. “The Blood‐Testis Barrier: Its Biology, Regulation, and Physiological Role in Spermatogenesis.” Current Topics in Developmental Biology 71: 263–296. 10.1016/s0070-2153(05)71008-5. 16344108

[mrd70012-bib-0053] Zhao, K. , Y. Liu , Z. Xiong , L. Hu , and C. Xiong . 2017. “Tissue‐Specific Inhibition of Urokinase‐Type Plasminogen Activator Expression in the Testes of Mice by Inducible Lentiviral RNA Interference Causes Male Infertility.” Reproduction, Fertility, and Development 29: 2149–2156. 10.1071/RD16477. 28298247

